# Aberrant White Matter Microstructure as a Potential Diagnostic Marker in Alzheimer's Disease by Automated Fiber Quantification

**DOI:** 10.3389/fnins.2020.570123

**Published:** 2020-09-24

**Authors:** Haifeng Chen, Xiaoning Sheng, Ruomeng Qin, Caimei Luo, Mengchun Li, Renyuan Liu, Bing Zhang, Yun Xu, Hui Zhao, Feng Bai

**Affiliations:** ^1^Department of Neurology, Drum Tower Hospital, Medical School of Nanjing University, Nanjing, China; ^2^The State Key Laboratory of Pharmaceutical Biotechnology, Institute of Brain Science, Nanjing University, Nanjing, China; ^3^Jiangsu Province Stroke Center for Diagnosis and Therapy, Nanjing, China; ^4^Nanjing Neuropsychiatry Clinic Medical Center, Nanjing, China; ^5^Department of Radiology, Drum Tower Hospital, Medical School of Nanjing University, Nanjing, China

**Keywords:** Alzheimer's disease, white matter microstructure, uncinate fasciculus, anterior thalamic radiation, cingulum cingulate

## Abstract

Neuroimaging evidence has suggested white matter microstructure are heavily affected in Alzheimer's disease (AD). However, whether white matter dysfunction is localized at the specific regions of fiber tracts and whether they would be a potential biomarker for AD remain unclear. By automated fiber quantification (AFQ), we applied diffusion tensor images from 25 healthy controls (HC), 24 amnestic mild cognitive impairment (aMCI) patients and 18 AD patients to create tract profiles along 16 major white matter fibers. We compared diffusion metrics [Fractional anisotropy (FA), mean diffusivity (MD), axial diffusivity (DA), and radial diffusivity (DR)] between groups. To assess the diagnostic value, we applied a random forest (RF) classifier, a type of machine learning method. In the global tract level, we found that aMCI and AD patients showed higher MD, DA, and DR values in some fiber tracts mostly in the left hemisphere compared to HC. In the point-wise level, widespread disruption were distributed on specific locations of different tracts. The point-wise MD measurements presented the best classification performance with respect to differentiating AD from HC. The two most important variables were localized in the prefrontal potion of left uncinate fasciculus and anterior thalamic radiation. In addition, the point-wise DA in the posterior component of the left cingulum cingulate displayed the most robust discriminative ability to identify AD from aMCI. Our findings provide evidence that white matter abnormalities based on the AFQ method could be as a diagnostic biomarker in AD.

## Introduction

Alzheimer's disease (AD), the most common type of dementia in the elderly, is a growing global public health concern with enormous implications for individuals, families and society (GBD 2016 Dementia Collaborators, 2019). Due to the distribution of its hallmark pathological changes [amyloid plaques (Aβ) and neurofibrillary tangles (NFT)], AD has traditionally been regarded as a disease of the brain's gray matter (Pini et al., 2016). In recent years, neuroimaging studies have suggested that in addition to the features of neuronal loss, white matter alterations may be the important pathophysiological characteristics of AD (Nasrabady et al., 2018). However, our knowledge about white matter degeneration in AD is still limited compared to what we know about gray matter atrophy (Nasrabady et al., 2018). In particular, whether the patterns of white matter changes are different across fiber tracts and whether they would be a promising biomarker for AD remain largely unknown.

Diffusion tensor imaging (DTI) has been a widely-used tool to detect microstructural integrity of white matter. Fractional anisotropy (FA) and mean diffusivity (MD) are two common quantitative metrics of DTI that detect the directionality and displacement of water diffusion. Several DTI analytical approaches, including regions of interest (ROI)-based analysis, voxel-based morphometry (VBM), and tract-based spatial statistics (TBSS) have been used in AD-related studies.

A cross-sectional study have reported that AD patients experienced significantly lower FA and higher MD in the regions of splenium and fornix compared with either normal elderly or mild cognitive impairment (MCI, the early stage of dementia) by the ROI-based method (Nowrangi et al., 2013). Results from VBM analysis have shown that AD patients with a high Braak NFT stage had significantly elevated MD values in the crus of fornix, precuneus, cingulum, and temporal white matter (Kantarci et al., 2017). Weiler et al. (2015) demonstrated that AD patients exhibited a progression of white matter degeneration over time involving the widespread white matter regions by applying TBSS. In fact, these research have not derived consistent results, which may be caused by different analytical methods.

However, we couldn't draw conclusion directly about disease-related white matter abnormalities by simply applying ROI-wise or voxel-wise comparison. The ROI-based method is subject to theoretical hypotheses of regions of pathologic damage, making localization difficult (Yeatman et al., 2011). Note that the VBM method does not have sufficient precision, particularly for patient populations at the individual level due to varied shapes of long-range fiber bundles among subjects (Yeatman et al., 2011). By contrast, TBSS, a skeleton-based approach, was proposed to reduce effects of local misregistration. Nevertheless, this method couldn't completely overcome the cross-subject co-registration problem (Goodrich-Hunsaker et al., 2018).

Furthermore, tissue diffusion properties may change along each tract because diseases can strike at different local positions within the bundle (Yeatman et al., 2012). Thus, an ideal method of the localization-specific properties along each fiber tract at the individual level may provide more detailed information about white matter abnormalities. Automated fiber quantification (AFQ) is a new analyzing method that applies a deterministic tractography approach to recreate whole-brain white matter tracts and estimate point-wise diffusion parameters aimed at the specific tract (Yeatman et al., 2012). Recently, AFQ has been successfully applied to research on other neuropsychiatric diseases (Deng et al., 2018; Huber et al., 2018; Chen et al., 2020). Consequently, AFQ may provide a promising strategy to investigate whether white matter microstructural integrity is abnormal along the entire tract or at the specific location on a tract in the progression of AD.

In this study, we aimed to utilize AFQ tractography method to explore the altered pattern of white matter fiber tracts across AD and amnestic MCI (aMCI, the prodromal stage of AD) compared with healthy controls (HC). Furthermore, we combined white matter diffusion metrics with a machine learning algorithm, random forest (RF), to make predictions for HC, aMCI, and AD at the individual level. We hypothesize that white matter disruption may vary along fiber tracts in different patterns, which is associated with AD-related cognitive impairment, and may provide potential candidate hallmarks for its early diagnosis.

## Materials and Methods

### Participants

Eighty-one elderly subjects (right-handed) participated in this retrospective study, including 26 aMCI patients, 30 AD patients and 25 HC. The aMCI and AD patients were recruited from the Memory Clinic of the Neurology Department, Nanjing Drum Tower Hospital and the demographically matched HC were recruited from the local community. This research was approved by the Ethics Committee of Nanjing Drum Tower Hospital, and informed consents were obtained from all participants.

All participants underwent a complete neurological evaluation, standard laboratory tests, neuroimaging examination, and an extensive battery of neuropsychological assessments. The possible or probable AD was diagnosed based on the National Institute of Neurological and Communicative Disorders and Stroke and the AD and Related Disorders Association (NINCDS-ADRDA) and the Diagnostic and Statistical Manual of Mental Disorders IV criteria (DSM-IV) guidelines (McKhann et al., 1984; American Psychiatric Association, 1994). The aMCI patients included in this study were diagnosed according to the recommendations of Petersen and described as follows (Petersen, 2004): (1) memory complaint confirmed by the subject and/or an informant; (2) objective cognitive performance documented by an auditory verbal learning test-delayed recall (AVLT-DR) scores below or equal to 1.5 SD of education- and age-adjusted norms; (3) clinical dementia rating (CDR) score = 0.5; (4) the scores for the Mini-Mental State Examination (MMSE) ≥ 24; and (5) not sufficient to dementia according to NINCDS-ADRDA and DSM-IV. More detailed information about the criteria of aMCI has been described in our previous study (Chen et al., 2018). The HC subjects were required to have MMSE scores ≥ 26 and CDR score of 0. In addition, participants with a history of other psychiatric or neurological disease (e.g., stroke, depression, traumatic brain injury, and others) were excluded in the current study.

### MRI Scanning

All of the subjects were examined on a 3.0T MRI scanner (Philips Medical Systems). The protocol included the diffusion-weighted imaging (DWI) sequence [echo time (TE) = 55 ms, repetition time (TR) = 9,154 ms, FOV = 224 × 224 mm^2^, acquisition matrix = 112 × 112, voxel size = 2 × 2 × 2.5 mm^3^, thickness = 2.5 mm, 32 gradient directions (b = 1,000 s/mm^2^) and 1 b0 image], the high-resolution T1-weighted imaging [TE = 4.6 ms, TR= 9.8 ms, flip angle (FA) = 8°, FOV = 250 × 250 mm^2^, acquisition matrix = 256 × 256, number of slices = 192, thickness = 1.0 mm] and the FLAIR sequence [TE = 333 ms, TR = 4,500 ms, time interval (TI) = 1,600 ms, voxel size = 0.95 × 0.95 × 0.95 mm^3^, number of slices = 200, acquisition matrix = 270 × 260].

### Neuropsychological Measurement

All participants underwent the standardized neuropsychological assessments performed by an experienced neuropsychologist (Liu et al., 2019). General cognitive functioning was evaluated by MMSE. The multiple cognition domains including episodic memory, executive function, language, information processing speed, and visuospatial function were also evaluated. The detailed neuropsychological battery consisted of AVLT-DR, Wechsler Memory Scale Visual Reproduction-delayed recall (WMS-VR-DR), Boston Naming Test (BNT), Category Verbal Fluency (CVF), Visual Reproduction-copy (VR-C), Clock Drawing Test (CDT), Stroop Color and Word Tests A, B, and C (Stroop A, B, and C) and Trail Making Test-A and -B (TMT-A and TMT-B). The raw scores were Z-transformed in order to calculate the composite score of each cognitive domain.

### Magnetic Resonance Image Pre-processing

DWI DICOM images were converted to NIfTI images using the dcm2nii tool in MRIcron (https://www.nitrc.org/projects/mricron), which automatically creates b-vector and b-value files. DTI data were then preprocessed using the the FMRIB Software Library (FSL) version 5.0.9 (https://www.fmrib.ox.ac.uk/fsl). First, the DWI volumes (b1000) were co-registered to the non-DWI image (b0) using an affine alignment. Then, DTI images were resampled to 2-mm isotropic voxels with eddy-current and motion correction using a rigid body alignment. Brain extraction tool (BET, in the FSL software) was used to remove the non-brain tissue. The diffusion tensor model was applied to estimate voxel-wise eigenvalues and eigenvectors and then to calculate the diffusion measures: FA, MD, axial diffusivity (DA), and radial diffusivity (DR) by DTIFIT tool (in the FSL software).

### Automated Fiber Quantification Procedure

We identified whole-brain white matter tracts (including 20 major fiber tracts), and further calculated the diffusion measurements along the tract fiber by utilizing the AFQ script (https://github.com/yeatmanlab/AFQ) (Yeatman et al., 2012). A brief description of the processing steps is as follows: (1) the deterministic tractography with the thresholds of FA > 0.2 and turning angle <30°; (2) the waypoint ROI-based tract identification as described in the previous research (Wakana et al., 2007); (3) the tract refinement based on the fiber probability maps (Hua et al., 2008); (4) fiber tract cleaning applying the outlier rejection algorithm (Yeatman et al., 2012); (5) quantification of the diffusion metrics along each fiber tract at 100 equidistant nodes. The identified 20 white matter tracts are listed in Table 1. Since AFQ uses the strict criterion for tract segmentation, it is possible not to identify all 20 fiber tracts in each subject (Johnson et al., 2014). We excluded 4 fiber tracts (the arcuate fasciculus and bilateral bilateral cingulum hippocampus) which were difficult to be identified than the others (Supplementary Table 1). Table 1 shows the detailed demographic and neuropsychological data of subjects in which AFQ identified all 16 tracts, and only these subjects (HC, *n* = 25; aMCI, *n* = 24; AD, *n* = 18) are into the further analyses.

**Table 1:** Demographic and neuropsychological data.

**Items**	**HC (*n =* 25)**	**aMCI (*n =* 24)**	**AD (*n =* 18)**	***F/χ2/T***	***p***	***Post hoc*** **analyses**		
						**HC vs. aMCI**	**HC vs. AD**	**aMCI vs. AD**
**Demographics**								
Age (years)	61.96 ± 6.52	67.08 ± 9.51	64.89 ± 8.06	2.463	0.093^b^	0.031^*^	0.247	0.389
Education (years)	11.44 ± 2.80	10.88 ± 2.97	9.28 ± 4.98	1.997	0.144^b^	0.581	0.054	0.155
Gender (male/female)	14/11	8/16	8/10	2.545	0.280^a^	0.111	0.455	0.463
**Vascular risk factors**								
Hypertension (%)	48	50	38.89	0.559	0.756	0.889	0.553	0.474
Diabetes (%)	40	37.5	44.44	0.208	0.901	0.858	0.771	0.650
Hypercholesterolemia (%)	32	29.17	27.78	0.097	0.952	0.830	0.766	0.921
**General cognition**								
MMSE	29.16 ± 0.85	27.46 ± 1.32	17.00 ± 6.14	79.128	<0.001^b*^	0.076	<0.001^*^	<0.001^*^
**Composition Z scores of each cognitive domain**					
Episodic Memory	0.65 ± 0.59	−0.67 ± 0.52	–	8.306	<0.001^c*^	–	–	–
AVLT-DR (raw data)	0.78 ± 0.65 (6.36 ± 1.63)	−0.82 ± 0.53 (2.38 ± 1.31)	–	9.402	<0.001^c*^	–	–	–
VR-DR (WMS) (raw data)	0.51 ± 0.96 (10.48 ± 4.81)	−0.53 ± 0.74 (5.25 ± 3.71)	–	4.252	<0.001^c*^	–	–	–
Information Processing Speed	0.32 ± 0.79	−0.34 ± 0.81	–	2.895	0.006^c*^	–	–	–
TMT-A (inverse) (raw data)	0.37 ± 0.94 (45.52 ± 12.56)	−0.38 ± 0.93 (65.63 ± 22.49)	–	2.822	0.003^c*^	–	–	–
Stroop A (inverse) (raw data)	0.34 ± 0.90 (17.72 ± 4.11)	−0.35 ± 0.99 (23.71 ± 10.74)	–	2.531	0.015^c*^	–	–	–
Stroop B (inverse) (raw data)	0.27 ± 0.85 (22.86 ± 9.79)	−0.28 ± 1.08 (31.00 ± 16.78)	–	1.985	0.053^c^	–	–	–
Language	0.27 ± 0.59	−0.29 ± 0.91	–	2.569	0.013^c*^	–	–	–
CVF (raw data)	0.23 ± 0.93 (16.80 ± 3.58)	−0.24 ± 1.03 (14.96 ± 3.99)	–	1.701	0.096^c^	–	–	–
BNT (raw data)	0.31 ± 0.62 (52.92 ± 5.61)	−0.33 ± 1.21 (47.13 ± 10.94)	–	2.346	0.023^c*^	–	–	–
Visuospatial Processing Function	0.36 ± 0.25	−0.37 ± 1.11	–	3.220	0.002^c*^	–	–	–
CDT (raw data)	0.43 ± 0.35 (3.96 ± 0.20)	−0.44 ± 1.25 (3.46 ± 0.72)	–	3.348	0.002^c*^	–	–	–
VR-C (raw data)	0.29 ± 0.39 (13.84 ± 0.55)	−0.30 ± 1.32 (13.00 ± 1.87)	–	2.156	0.036^c*^	–	–	–
Executive Function	0.34 ± 0.79	−0.35 ± 0.69	–	3.256	0.002^c*^	–	–	–
TMT-B (inverse) (raw data)	0.41 ± 1.05 (100.44 ± 54.41)	−0.42 ± 0.76 (156.13 ± 80.47)	–	3.154	0.003^c*^	–	–	–
Stroop C (inverse) (raw data)	0.27 ± 0.90 (36.23 ± 12.66)	−0.28 ± 1.04 (54.71 ± 39.15)	–	1.978	0.054^c^	–	–	–

*Values are presented as the mean ± standard deviation (SD) or percentages*.

a *the p-value was obtained by χ2 test*,

b *the p-value was obtained by one-way ANOVA*,

c *the p-value was obtained by two sample t-test*.

* *indicates a statistical difference between groups, p < 0.05*.

*HC, health control; aMCI, amnestic mild cognitive impairment; AD, Alzheimer's Disease; MMSE, mini mental state examination; AVLT-DR, Auditory Verbal Learning Test-delayed recall; VR-DR, visual reproduction-delay recall; WMS, Wechsler Memory Scale; CVF, category verbal fluency; BNT, Boston Naming Test; CDT, Clock Drawing Test; VR-C, visual reproduction-copy; TMT-A and TMT-B, Trail Making Test-A and B; Stroop A, B and C, Stroop Color and Word Tests A, B, and C*.

### Statistical and Machine Learning Analyses

Demographic characteristics (e.g., age, gender, and years of education), vascular risk factors (hypertension, diabetes, and hypercholesterolemia) and cognitive performance were compared using the Chi-squared (χ2) test, two-sample *t-*test or one-way analysis of variance (ANOVA) in SPSS software Version 22 (IBM Corp., Armonk, New York). The significant threshold was set at *P* < 0.05.

To examine between-group difference in the global tract level, we calculated DTI measurements of 16 fiber tracts and performed the ANOVA. Age, gender and years of education were controlled as confounding covariates.

Then, the point-wise analyses were conducted by “Randomize” command of FSL software. Age, gender and years of education were included as confounding covariates in the general linear model (GLM). The false discovery rate (FDR) correction was used to determine the significance for *p*-values (*p* < 0.05) and only ≥ 3 adjacent nodes were reported (Banfi et al., 2019). To explore the relationship between white matter microstructural integrity and cognition, partial correlation analyses were performed by SPSS while controlling for age, gender, and years of education.

Finally, we applied the random forest (RF) classifier to identify the white matter diffusion metrics that best predicted the disorder diagnosis. Only those features significantly different between groups were considered in this analysis. In our research, RF was constructed with 500 trees (Numan et al., 2017). Every decision tree was built by a bootstrap sample from the training data. The out-of-bag (OOB) performance was predicted to obtain the unbiased estimation of the misclassification. Then, the RF framework estimated the importance of a variable by seeking how much the classification errors increased when the OOB data for that variable were permuted while all others were left unchanged. Furthermore, we ranked the variables' importance by assigning a score to each covariate based on the ability to classify correctly the dependent label. Due to the limited number of included subjects, we applied the leave-one-out cross-validation to assess the classification performance. The diagnostic ability of the neuroimaging features was evaluated according to accuracy, sensitivity and specificity. Accuracy is defined as (TP + TN)/(TP + TN + FN + FP), sensitivity is defined as TP/(TP + FN) and specificity is defined as TN/(FP + TN), where TN is the number of true negatives, TP is the number of true positives, FN is the number of false negatives, and FP is the number of false positives.

## Results

### Demographic and Clinical Characteristics

Demographic and clinical information for the HC, aMCI and AD in which AFQ successfully identified all 16 fiber tracts were provided in Table 1. There was no significant difference for age, gender distribution, years of education and vascular risk factors between three subgroups (*p* > 0.05). The AD patients showed poorer performances on MMSE (*p* < 0.001) than HC and aMCI. Because the AD patients didn't cooperate or understand the questions, some cognitive domain assessment scores were missing. Thus, we mainly compared the multiple cognitive domain performance between HC and aMCI. The aMCI group exhibited significantly lower scores in information processing speed (*p* < 0.011), episodic memory (*p* < 0.001), language (*p* < 0.013), visuospatial processing function(*p* < 0.002), and executive function (*p* < 0.002) than HC group.

### Between-Group Difference in Diffusion Metrics and Its Relationship With Cognition

Between-group difference in the global fiber tracts and point-wise level was determined by diffusion metrics (FA, MD, DA, and DR) with AFQ.

**FA**. No significant between-group difference was found in the fiber tract level (ANOVA, *p* > 0.05; Supplementary Table 2) and point-wise level (FDR correction, *p* > 0.05).

**MD**. In the fiber tract level, the left anterior thalamic radiation (ATR), ILF, corticospinal tract (CST), cingulum cingulate (CC), inferior fronto-occipital fasciculus (IFOF), superior longitudinal fasciculus (SLF), and forceps minor exhibited the significant difference between HC, aMCI, and AD (Table 2). The further analysis indicated that the AD patients showed significantly increased MD in these fiber tracts compared to HC and aMCI group. However, no significant alteration was observed between HC and aMCI.

**Table 2:** Mean MD (× 100) of 16 fiber tracts for HC, aMCI, and AD.

**Index**	**Tract**	**Group**			***F***	***p***	***Post hoc*** **analyses**		
		**HC**	**aMCI**	**AD**			**HC vs. aMCI**	**HC vs. AD**	**aMCI vs. AD**
1	ATR_L	72.60 ± 2.53	74.83 ± 4.75	77.38 ± 6.76	4.687	0.013^*^	0.393	0.004^*^	0.033^*^
2	ATR_R	72.53 ± 2.81	74.74 ± 4.92	76.10 ± 6.09	2.042	0.139	–	–	–
3	CST_L	73.11 ± 2.40	73.96 ± 2.41	75.37 ± 2.77	3.748	0.029^*^	0.718	0.013^*^	0.031^*^
4	CST_R	73.25 ± 2.20	74.67 ± 2.41	75.25 ± 3.01	2.172	0.123	–	–	–
5	CC_L	76.76 ± 2.57	77.05 ± 4.09	80.04 ± 3.36	7.469	0.001^*^	0.758	0.001^*^	0.002^*^
6	CC_R	75.02 ± 2.59	76.02 ± 3.57	76.69 ± 4.09	1.431	0.247	–	–	–
7	Forceps major	90.61 ± 6.81	93.05 ± 9.69	93.27 ± 8.54	0.435	0.649	–	–	–
8	Forceps minor	79.49 ± 3.86	81.98 ± 4.95	84.10 ± 4.14	5.520	0.006^*^	0.180	0.002^*^	0.047^*^
9	IFOF_L	80.25 ± 2.49	81.98 ± 5.32	84.62 ± 4.17	5.607	0.006^*^	0.287	0.002^*^	0.026^*^
10	IFOF_R	79.78 ± 2.74	79.93 ± 4.45	82.37 ± 6.17	2.849	0.066	–	–	–
11	ILF_L	81.32 ± 2.45	81.30 ± 5.13	84.39 ± 4.60	5.211	0.008^*^	0.961	0.006^*^	0.006^*^
12	ILF_R	79.17 ± 3.09	79.56 ± 4.13	78.75 ± 4.83	0.074	0.929	–	–	–
13	SLF_L	73.04 ± 2.62	73.34 ± 3.00	75.08 ± 3.47	3.557	0.035^*^	0.810	0.017^*^	0.030^*^
14	SLF_R	73.65 ± 2.17	73.90 ± 3.66	75.42 ± 3.40	1.621	0.206	–	–	–
15	UF_L	79.18 ± 2.55	80.42 ± 3.04	81.13 ± 3.21	2.948	0.060	–	–	–
16	UF_R	78.57 ± 2.12	78.50 ± 2.94	79.49 ± 3.60	0.632	0.535	–	–	–

*Values are presented as the mean ± standard deviation (SD)*.

* *indicates a statistical difference between groups, p < 0.05*.

*HC, health control; aMCI, amnestic mild cognitive impairment; AD, Alzheimer's Disease; MD, mean diffusivity; ATR, anterior thalamic radiation; CST, corticospinal tract; CC, cingulum cingulate; IFOF, inferior fronto-occipital fasciculus; ILF, inferior longitudinal fasciculus; SLF, superior longitudinal fasciculus; UF, uncinate fasciculus; R, right; L, left*.

**Figure 1:**
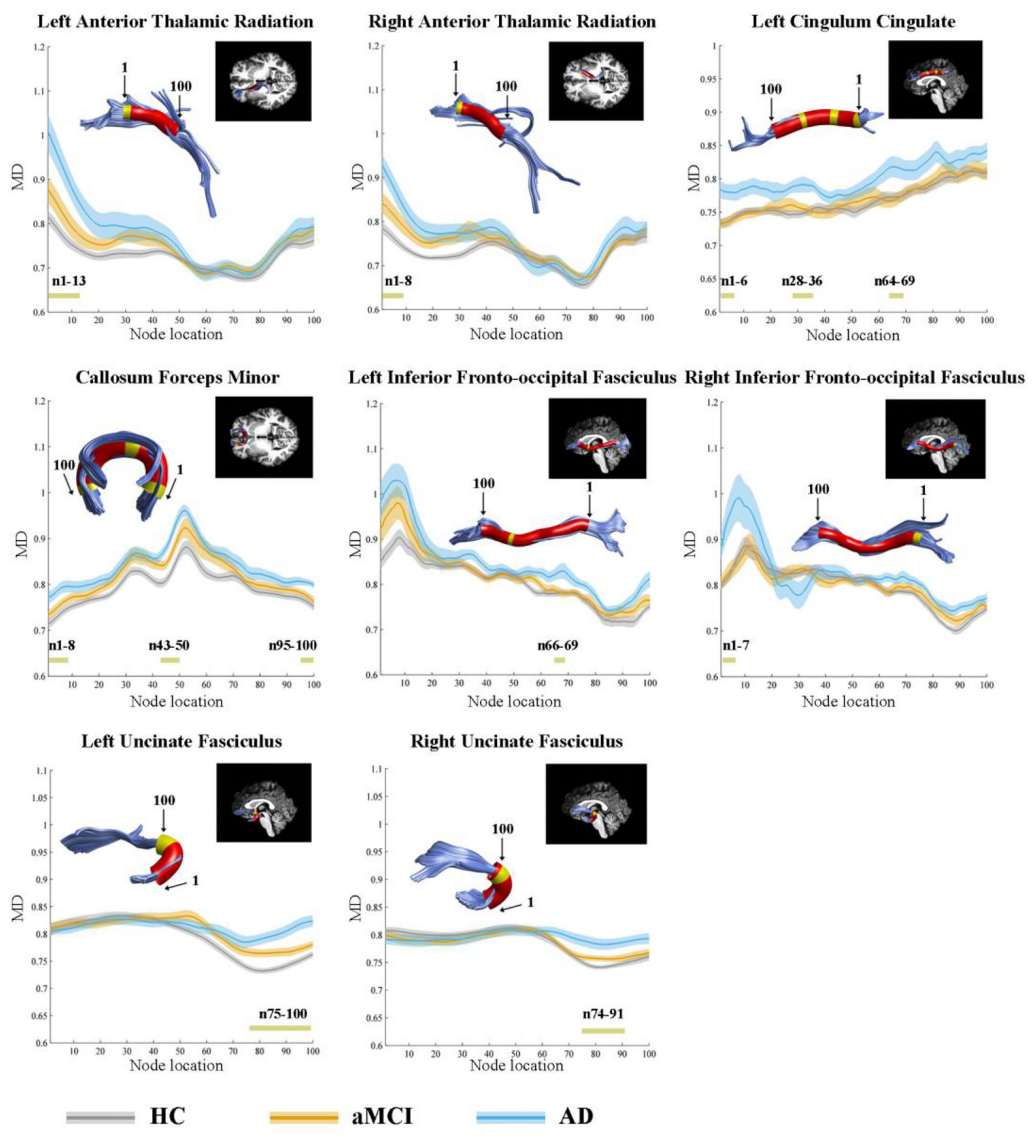
Significantly altered MD values in point-wise of fiber tracts (FDR correction, *p* < 0.05). Yellow color in the white matter tracts represents significantly altered locations. Red color in the white matter tracts represents other locations with no statistical significance. HC, health control; aMCI, amnestic mild cognitive impairment; AD, Alzheimer's Disease; MD, mean diffusivity.

In the point-wise comparison, significantly altered locations of fiber tracts (FDR correction, *p* < 0.05) were as follows: (1) the anterior component of the left ATR (nodes 1–13); (2) the anterior component of the right ATR (nodes 1–8); (3) the intermediate and posterior component of the left CC (nodes 1–6; nodes 28–36; nodes 64–69); (4) the widespread distribution of the forceps minor (nodes 1–8; nodes 43–50; nodes 95–100); (5) the anterior component of the left IFOF (nodes 66–69); (6) the posterior component of the right IFOF (nodes 1–7); (7) the frontal lobe portion of the left uncinate fasciculus (UF, nodes 75–100); (8) the frontal lobe portion of the right UF (nodes 74–91) (Figure 1). The further *post-hoc* comparisons were showed in Table 3. In the point-wise level, the AD patients showed significantly increased MD in these locations of the fiber tracts compared to HC and aMCI group. It should be noted that aMCI obtained significantly higher mean MD values compared to HC in the frontal lobe portion of the left UF (nodes 75–100).

**Table 3:** The location of tracts showing significant group differences in diffusion measures (× 100) between HC, aMCI, and AD.

**Diffusion measures/fiber tracts**	**Location**	**Group**			***F***	***p***	***Post hoc*** **analyses**		
		**HC**	**aMCI**	**AD**			**HC vs. aMCI**	**HC vs. AD**	**aMCI vs. AD**
**FA**									
– –	–	–	–	–	–	–	–	–	–
**MD**									
ATR_L	n1–13	77.33 ± 6.44	82.19 ± 12.84	92.39 ± 13.88	8.958	<0.001^*^	0.502	<0.001^*^	0.001^*^
ATR_R	n1–8	76.74 ± 5.69	82.08 ± 10.73	88.70 ± 10.77	8.284	0.001^*^	0.479	<0.001^*^	0.002^*^
CC_L	n1–6	73.59 ± 3.01	73.51 ± 3.94	78.12 ± 5.16	10.457	<0.001^*^	0.819	<0.001^*^	<0.001^*^
	n28–36	74.95 ± 2.82	75.51 ± 4.27	78.98 ± 4.13	7.754	0.001^*^	0.619	<0.001^*^	0.002^*^
	n64–69	77.29 ± 3.96	77.03 ± 4.58	81.68 ± 6.30	7.821	0.001^*^	0.938	0.001^*^	<0.001^*^
Forceps minor	n1–8	73.04 ± 4.60	74.99 ± 4.94	78.53 ± 5.49	8.212	0.001^*^	0.235	<0.001^*^	0.006^*^
	n43–50	82.78 ± 5.36	86.70 ± 6.08	93.32 ± 4.95	8.791	<0.001^*^	0.071	<0.001^*^	0.017^*^
	n95–100	76.09 ± 4.08	77.34 ± 4.16	80.35 ± 2.93	7.829	0.001^*^	0.480	<0.001^*^	0.003^*^
IFOF_L	n66–69	78.11 ± 2.85	78.16 ± 4.61	82.72 ± 6.95	6.116	0.004^*^	0.915	0.003^*^	0.003^*^
IFOF_R	n1–7	82.45 ± 5.67	82.61 ± 8.98	94.46 ± 17.47	8.057	0.001^*^	0.864	0.001^*^	0.001^*^
UF_L	n75–100	74.17 ± 2.48	76.84 ± 3.50	80.26 ± 4.04	19.525	<0.001^*^	0.012^*^	<0.001^*^	<0.001^*^
UF_R	n74–91	74.51 ± 2.04	75.75 ± 2.63	78.52 ± 4.09	9.134	<0.001^*^	0.197	<0.001^*^	0.004^*^
**DA**									
ATR_L	n1–27	105.34 ± 6.63	109.37 ± 11.84	118.72 ± 12.60	8.626	0.001^*^	0.642	<0.001^*^	0.001^*^
ATR_R	n1–9	103.45 ± 8.55	109.19 ± 13.19	117.58 ± 13.36	7.801	0.001^*^	0.621	<0.001^*^	0.002^*^
CST_L	n71–100	119.19 ± 5.06	121.66 ± 7.59	128.14 ± 7.83	10.437	<0.001^*^	0.234	<0.001^*^	0.002^*^
CST_R	n80–88	124.28 ± 6.75	125.18 ± 9.00	133.38 ± 8.72	7.018	0.002^*^	0.885	0.001^*^	0.002^*^
	n92–100	111.65 ± 4.63	111.55 ± 7.04	119.00 ± 6.54	8.358	0.001^*^	0.960	0.001^*^	0.001^*^
CC_L	n1–10	107.39 ± 7.61	109.96 ± 6.20	117.53 ± 5.47	11.942	<0.001^*^	0.149	<0.001^*^	0.001^*^
Forceps minor	n1–12	103.66 ± 6.17	106.88 ± 8.18	111.78 ± 8.29	8.314	0.001^*^	0.245	<0.001^*^	0.006^*^
	n45–53	162.48 ± 8.63	168.33 ± 10.96	174.07 ± 8.10	8.185	0.001^*^	0.093	<0.001^*^	0.019^*^
	n92–100	107.84 ± 5.77	109.87 ± 5.65	114.51 ± 4.23	10.273	<0.001^*^	0.604	<0.001^*^	<0.001^*^
IFOF_L	n5–10	129.54 ± 12.59	142.20 ± 22.50	149.85 ± 20.96	6.530	0.003^*^	0.012^*^	0.001^*^	0.318
	n65–69	121.78 ± 4.96	124.16 ± 6.73	130.59 ± 9.10	9.114	<0.001^*^	0.166	<0.001^*^	0.005^*^
	n93–100	110.03 ± 4.68	112.32 ± 8.48	120.72 ± 8.07	12.494	<0.001^*^	0.511	<0.001^*^	<0.001^*^
IFOF_R	n85–89	104.60 ± 5.20	107.40 ± 8.10	112.30 ± 6.19	6.683	0.002^*^	0.521	0.001^*^	0.006^*^
UF_L	n75–91	117.88 ± 8.27	122.79 ± 6.30	127.87 ± 9.78	7.665	0.001^*^	0.051	<0.001^*^	0.048^*^
**DR**									
ATR_L	n1–9	64.08 ± 6.85	69.30 ± 12.13	80.07 ± 14.44	9.977	<0.001^*^	0.427	<0.001^*^	0.001^*^
ATR_R	n1–3	65.01 ± 6.37	70.38 ± 10.52	77.26 ± 10.19	8.501	0.001^*^	0.419	<0.001^*^	0.002^*^
CC_L	n77–81	60.42 ± 4.92	61.72 ± 5.36	67.00 ± 7.16	7.802	0.001^*^	0.428	<0.001^*^	0.004^*^
IFOF_R	n1–6	59.87 ± 5.34	61.02 ± 9.80	74.32 ± 16.86	11.504	<0.001^*^	0.834	<0.001^*^	<0.001^*^
UF_L	n91–100	51.78 ± 3.15	54.05 ± 4.78	57.28 ± 4.66	11.161	<0.001^*^	0.058	<0.001^*^	0.006^*^
UF_R	n78–91	50.85 ± 3.25	52.84 ± 3.33	55.98 ± 4.03	10.303	<0.001^*^	0.084	<0.001^*^	0.006^*^

*Values are presented as the mean ± standard deviation (SD)*.

* *indicates a statistical difference between groups, p < 0.05*.

*HC, health control; aMCI, amnestic mild cognitive impairment; AD, Alzheimer's Disease; FA, fractional anisotropy; MD, mean diffusivity; DA, axial diffusivity; DR, radial diffusivity; ATR, left anterior thalamic radiation; CST, corticospinal tract; CC, cingulum cingulate; IFOF, inferior fronto-occipital fasciculus; UF, uncinate fasciculus; R, right; L, left*.

**DA**. Supplementary Table 3 showed DA values of each fiber tract. The left ATR, bilateral CST, forceps minor, left IFOF, left ILF, left SLF, and left UF displayed significant differences between HC, aMCI, and AD. The further *post-hoc* analyses were also listed in Supplementary Table 3.

Significant alterations in the point-wise comparison (FDR correction, *p* < 0.05) were mainly located in these tracts as follows: (1) the anterior component of the left ATR (nodes 1–27); (2) the anterior component of the right ATR (nodes 1–9); (3) the superior portion of the left CST (nodes 71–100); (4) the superior portion of the right CST (nodes 80–88; nodes 92–100); (5) the posterior component of the left CC (nodes 1–10); (6) the widespread distribution of the forceps minor (nodes 1–12; nodes 43–53; nodes 92–100); (7) the anterior and posterior component of the left IFOF (nodes 5–10; nodes 65–69; nodes 93–100); (8) the anterior component of the right IFOF (nodes 85–89); (9) the frontal lobe portion of the left UF (nodes 75–91) (Supplementary Figure 1). The further *post-hoc* comparisons were listed in Table 3.

**DR**. **Supplementary Table 4** showed that the three groups exhibited significant differences in the left CC and left ILF. The further multiple comparisons were showed in Supplementary Table 4.

In point-wise comparison, significantly altered locations of fiber tracts (FDR correction, *p* < 0.05) were as follows: (1) the anterior component of the left ATR (nodes 1–9); (2) the anterior component of the right ATR (nodes 1–3); (3) the anterior component of the left CC (nodes 77–81); (4) the posterior component of the right IFOF (nodes 1–6); (5) the frontal lobe portion of the left UF (nodes 91–100); (6) the frontal lobe portion of the right UF (nodes 78–91) (Supplementary Figure 2). The further *post-hoc* analyses were showed in Table 3.

For the altered fiber tracts and point-wise location, we found no significant correlation between diffusion metrics and cognitive performance in aMCI or AD.

### Discrimination Analysis

**Figure 2:**
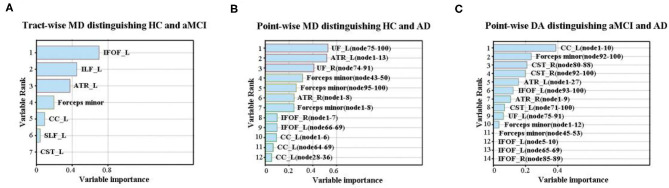
The variable importance of disease diagnosis. **(A)** The tract-level MD profile distinguished aMCI from HC with discrimination ability (accuracy = 75.51%; sensitivity = 80.00%; specificity = 80.83%). The left IFOF was the most important tract contributing to HC *VS*. aMCI classification. **(B)** With respect to differentiating AD from HC, the point-wise MD measurements presented the best classification performance (accuracy = 86.05%; sensitivity = 92.00%; specificity = 77.78%). The two most important variables were localized mainly in tracts along the left UF and ATR. **(C)** The point-wise DA measurements distinguished AD from aMCI with discrimination ability (accuracy = 77.78%; sensitivity = 66.67%; specificity = 61.11%), where the posterior component of the left CC was the most important variable. HC, health control; aMCI, amnestic mild cognitive impairment; AD, Alzheimer's Disease; MD, mean diffusivity; DA, axial diffusivity; ATR, anterior thalamic radiation; CST, corticospinal tract; CC, cingulum cingulate; IFOF, inferior fronto-occipital fasciculus; ILF, inferior longitudinal fasciculus; SLF, superior longitudinal fasciculus; UF, uncinate fasciculus; R, right; L, left.

Table 4 showed the results of the RF for the disorder diagnosis based on diffusion metrics in the fiber tract level and point-wise level. Among these results, we only focused on the classification where accuracy was over 70%. The tract-level MD profile could be applied to distinguish aMCI from HC with the highest discrimination ability (accuracy = 75.51%; sensitivity = 80.00%; specificity = 80.83%). Furthermore, the analysis of the variable importance showed that the left IFOF was the most important tract contributing to HC *VS*. aMCI classification (Figure 2A). With respect to differentiating AD from HC, the point-wise MD measurements presented the best classification performance (accuracy = 86.05%; sensitivity = 92.00%; specificity = 77.78%). The two most important variables were localized mainly in tracts along the left UF (nodes 75–100) and left ATR (nodes 1–13) (Figure 2B). In addition, the point-wise DA measurements had a significant role in the aMCI *VS*. AD classification (accuracy = 77.78%; sensitivity = 66.67%; specificity = 61.11%), where the posterior component of the left CC (nodes 1–10) was the most important variable (Figure 2C). Other results about the variable importance differentiating AD from HC was presented in Supplementary Figure 3.

**Table 4:** Discrimination results derived from the RF between HC, aMCI, and AD.

**Diffusion measures**		**HC vs. aMCI**			**HC vs. AD**			**aMCI vs. AD**		
		**Accuracy**	**Specificity**	**Sensitivity**	**Accuracy**	**Specificity**	**Sensitivity**	**Accuracy**	**Specificity**	**Sensitivity**
FA	–	–	–	–	–	–	–	–	–	–
MD	Tract level	**75.51%**	**80.00%**	**80.83%**	**76.74%**	**84.00%**	**66.67%**	52.38%	54.17%	50.00%
	Point-wise level	67.36%	68.00%	66.67%	**86.05%**	**92.00%**	**77.78%**	61.90%	70.83%	50.00%
DA	Tract level	61.22%	56.00%	66.67%	**81.40%**	**84.00%**	**77.78%**	66.67%	75.00%	55.56%
	Point-wise level	53.06%	52.00%	54.17%	**83.72%**	**88.00%**	**83.33%**	**77.78%**	**66.67%**	**61.11%**
DR	Tract level	69.39%	64.00%	75.00%	60.47%	72.00%	44.44%	50.00%	66.67%	27.78%
	Point-wise level	63.27%	68.00%	58.33%	**81.40%**	**88.00%**	**72.22%**	59.52%	66.67%	50.00%

*HC, health control; aMCI, amnestic mild cognitive impairment; AD, Alzheimer's Disease; RF, random forest; FA, fractional anisotropy; MD, mean diffusivity; DA, axial diffusivity; DR, radial diffusivity. The accuracy more than 70% were in bold*.

## Discussion

In this study, we applied a precise method of fiber-tract segmentation (AFQ) to identify the localized significance of aberrant white matter microstructure and further investigate their role in the diagnosis of AD. To be noted, we found the following points: (1) The left-sided microstructural vulnerability in the white matter fiber tract level in AD; (2) The frontal lobe portion of left UF and ATR playing the critical role in the diagnosis of AD; (3) The posterior component of the left CC may be as a neuroimaging marker of monitoring disease progression.

In our research, only DA, DR, and MD values of white matter in the entire fiber tract or point-wise level exhibited the significant alterations. The DA indicates the diffusion coefficient parallel to the principal eigenvector and DR reflects the average diffusivity along the secondary axes, whereas MD is calculated as an average rate of all three diffusion axes. Higher MD values often imply impaired white matter integrity (Bennett and Madden, 2014). It has been proposed that DA is more sensitive to axonal damage while RD is more sensitive to myelin loss (Bennett and Madden, 2014). However, no significant alterations in FA (an index of the directionality of water diffusion) along fiber tracts were observed in our study. As reviewed by Amlien and Fjell, the increased DA could contribute to reduce between-group differences in FA, and MD and DR may thus be as more sensitive neuroimaging biomarkers of white matter damage in AD (Amlien and Fjell, 2014). This similar pattern has also been demonstrated by a cross-sectional and longitudinal study, which revealed that increased MD and DA were the first abnormalities to occur in the early phase of AD (Acosta-Cabronero et al., 2012).

### The Leftward White Matter Microstructural Vulnerability in the Fiber Tract Level

In the fiber tract level, these significantly altered fiber tracts (e.g., the left IFOF, ATR, CC, ILF, SLF, UF, and CST) were mostly located in the left hemispheric. Previous studies have revealed that AD may appear to begin with or be characterized by the asymmetric left lateralized pattern of neurodegeneration. Earlier researches concerning brain metabolism in AD or individuals at risk for AD showed asymmetric cerebral glucose metabolic deficits detected with fludeoxyglucose F18, predominantly in parts with left hemispheric hypometabolism (Loewenstein et al., 1989; Small et al., 1995). Recently, the longitudinal analysis using fludeoxyglucose positron emission tomography further demonstrated that Aβ positive patients with MCI indicated regional brain metabolic rate of glucose declines mainly located in the left temporal cortex, correlated with cognitive decline (Weise et al., 2018). From the perspective of brain morphometry, asymmetric patterns of brain atrophy with a left lateralized pronunciation (particularly located in the left parietal and temporal lobe) have been reported in early stage of AD. As the disease progresses, gray matter loss becomes more symmetrical (Janke et al., 2001). In addition, a meta-analyses focusing on hippocampal volume determined a left-less-than-right atrophy pattern in MCI and AD comparing to normal aging and MCI patients shown the strongest effect sizes (Shi et al., 2009). However, previous DTI studies based on TBSS or VBM haven't yielded the consistent result, possibly due to the difference of analytical approach. So far, no clear-cut mechanism has been proposed to explain why left hemispheric fiber tracts could be more susceptible in the progression of AD. Specific focus was given to the amyloid deposition showing a left laterality (mainly in dorsal frontal cortex and sensory-motor area) in MCI subjects (Raji et al., 2008). Combining the white matter damage hypothesis in AD mentioned earlier, we infer that the left lateralized gray matter atrophy and amyloid deposition have a synergistic effect with the leftward white matter microstructural vulnerability in the fiber tract level.

It should be noted that the highest discrimination ability in identifying aMCI or AD from the normal elderly was obtained by the diffusion metrics of left IFOF among white matter tractography measures. The IFOF is a long cortico-cortical association tract that connects the orbitofrontal, posterior temporal, and the occipital areas. Remarkably, the left IFOF has been regarded as the ventral pathway involving semantic processing and executive function (Epstein et al., 2014; Sierpowska et al., 2019). Analysis of the white matter microstructure in preclinical phases of AD has revealed that the cognitively normal elderly with positive Aβ-42 show increased DA in the left IFOF compared to the aged with negative Aβ-42 (Molinuevo et al., 2014). Moreover, the macroscopic research of white matter injury in AD also provided the evidence that the volume of white matter hyperintensity within IFOF was the highest among all fiber tracts and correlated with decreased functional connectivity in IFOF-connected areas of default mode network (Taylor et al., 2017). Therefore, the above findings indicated that left-sided white matter microstructural integrity in the fiber tract level may be more vulnerable and the left IFOF may be a potential biomarker for the early identification of AD.

### The Frontal Lobe Portion of Left UF and ATR Playing the Important Value in the Diagnosis of AD

The AFQ technique can provide more information about white matter damage which is not obvious from the entire fiber bundles because the tissue properties of a fiber tract may change along the trajectory of the bundle. In point-wise comparison of MD profiles, twelve segmental components of eight fiber tracts displayed significant increased MD values in AD patients compared to HC. These altered metrics showed the best classification performance (accuracy = 86.05%) with respect to differentiating AD from HC. Moreover, the frontal lobe portion of left UF and ATR play the most critical role in the identification of AD.

The UF is a ventral long-range fiber tract that connects the lateral orbitofrontal cortex with the anterior temporal lobe, and is correlated with the formation and retrieval of episodic memory and other cognition (Olson et al., 2015). A meta-analysis examining white matter microstructural disruption concluded that a large effect size was detected in the UF, while a small effect size was showed in occipital white matter in the comparison of AD with normal aged (Sexton et al., 2011). According to the retrogenesis model, the white matter deterioration in AD is the reverse of processing of myelogenesis (Rubial-Álvarez et al., 2013). Early-myelinated projection fibers such as the corticospinal tracts are affected last and least, whereas late-myelinated cortico-cortical association tracts are affected early in AD (Rubial-Álvarez et al., 2013). The UF is one of the latest myelinated fasciculus, and continues maturing until the third decade of life (Olson et al., 2015). This might help explain why the UF is more susceptible to degeneration in AD. However, previous DTI studies have reported inconsistent findings in AD with some researches suggesting relatively lower FA and higher MD in the left UF (Molinuevo et al., 2014) while others have revealed relatively lower FA and higher MD in the right UF (Vipin et al., 2019) or in the bilateral UF (Rémy et al., 2015; Mayo et al., 2016). Our results further demonstrated that AD patients exhibited the increased MD in the frontal lobe portion of bilateral UF rather than in the entire fiber tract of UF and the frontal lobe portion of left UF in diagnosis of AD was relatively more important. The potential pathophysiological mechanism of the vulnerability in the frontal lobe portion of the left UF still needs to be investigated by further studies.

Another specific anatomical localization of the fiber tract contributing to determining AD patients from the elderly is the frontal lobe portion of left ATR. The ATR consists of nerve fibers connecting anterior and mediodorsal thalamic nuclei to the anterior cingulate cortex and prefrontal cortex, and has been regarded as the important structural pathway of cognition and behavior (Mamah et al., 2010). Our result is partially consistent with a previous study in which the individuals with subjective cognitive impairment have presented disrupted white matter integrity in the left ATR, indicating that the left ATR may serve as early marker of AD spectrum (Shao et al., 2019). Additionally, the macroscopic white matter research found a strict relationship between the presence of white matter lesions in the ATR and the severity of apathy (a risk factor of conversion to dementia) in aMCI (Torso et al., 2015). The imaging genetics studies suggested that the genetic variant (e.g., superoxide dismutase 2 and catalase genes and the human neuregulin-1 gene) may mediate cognitive and behavioral abnormality through their effects on brain structure in the ATR (Barnes et al., 2012; Salminen et al., 2017). The evidence that Aβ and NFT deposition preferentially occurred in the prefrontal may provide the potential mechanism of the ATR's prefrontal portion as a diagnostic biomarker (Lo et al., 2013; Tsartsalis et al., 2018).

### The Posterior Component of the Left CC May Be as a Neuroimaging Marker of Monitoring Disease Progression

Sexton et al. reviewed AD-related DTI studies and proposed that integrity difference varied along the CC and the largest effect size was in the posterior portion (Sexton et al., 2011). This pattern was similar to our result that the point-wise DA measurements had a significant role in differentiating the AD from aMCI, where the posterior component of the left CC was the most important variable. Although the left IFOF, UF and ATR played the critical role in identifying aMCI or AD from normal controls as mentioned above, the posterior component of left CC was relatively more important in the aspect of monitoring disease progression. The neuronal cell bodies of the CC are located mainly in the posterior cingulate cortex (PCC) and the posterior component of the CC is adjacent to the PCC. Despite structural neuroimaging researches have consistently suggested the earliest gray matter atrophy in the medial temporal lobe (e.g., entorhinal cortex and hippocampus) in AD spectrum (Moscoso et al., 2019), functional imaging studies have shown that hypoperfusion or hypometabolism first occur in the PCC (Teipel et al., 2016). The PCC functional deficit has been hypothetically explained by direct effect of AD-related pathological deposition and PCC atrophy and the indirect effect of medial temporal lobe atrophy (Choo et al., 2010). Thus, according to the Wallerian-like degeneration, the posterior CC dysfunction may be secondary to PCC functional deficit in AD (Choo et al., 2010). It still needs additional confirmation by longitudinal follow-up studies.

There were several potential limitations to this research. First, these observations in our cross-sectional study were made in a relatively small sample and still require further investigation by prospective studies on large populations. Second, the AD and aMCI diagnosis was not based on AD-related biomarkers but only the neuropsychological assessments. This might cause imprecise diagnosis. Third, due to the strict criterion in the fiber tracking, the fiber tracts like bilateral arcuate fasciculus and bilateral cingulum hippocampus tracts were failed to track in some subjects. Fourth, due to the internal associations among MD, DA and DR, we did not combine these measurements to assess the classification performance, which need to be further investigated. Fifth, we did not explore the effect of vascular risk factors on our result. Finally, association analyses showed that cognitive performance in aMCI or AD was not correlated with any of the significantly changed DTI metrics. This might result from our small sample size and the loss of multiple neuropsychological scores in AD patients.

## Conclusion

Combining a tractography approach with RF analyses, our study indicated abnormal tissue diffusion measures of global and local fiber tracts and their powerful diagnostic ability in aMCI and AD patients. We found that the left-sided microstructural integrity was vulnerable in the white matter fiber tract level in AD. Furthermore, the frontal lobe portion of left UF and ATR and the posterior component of the left CC played the critical role in the diagnosis and surveillance of AD. Our findings lent support to the viewpoint of white matter abnormalities as a diagnostic biomarker and provided deeper understanding of white matter changes in AD.

## Data Availability Statement

The raw data supporting the conclusions of this article will be made available by the authors, without undue reservation.

## Ethics Statement

The studies involving human participants were reviewed and approved by the Ethics Committee of Nanjing Drum Tower Hospital. The patients/participants provided their written informed consent to participate in this study.

## Author Contributions

FB and HZ designed this study. HC wrote the manuscript. RQ, XS, CL, ML, RL, and BZ collected the data. HC and RQ analyzed the data. FB, HZ, and YX revised the manuscript. All authors contributed to the article and approved the submitted version.

## Conflict of Interest

The authors declare that the research was conducted in the absence of any commercial or financial relationships that could be construed as a potential conflict of interest.
