# Supplementary material for: Aberrant White Matter Microstructure as a Potential Diagnostic Marker in Alzheimer's Disease by Automated Fiber Quantification
Source: Front Neurosci. 2020 Sep 24;14:570123. doi: 10.3389/fnins.2020.570123 (PMC7541946; doi:10.3389/fnins.2020.570123)
Supplement: Supplementary file 1 [file Data_Sheet_1.pdf]

Supplementary Figure 1

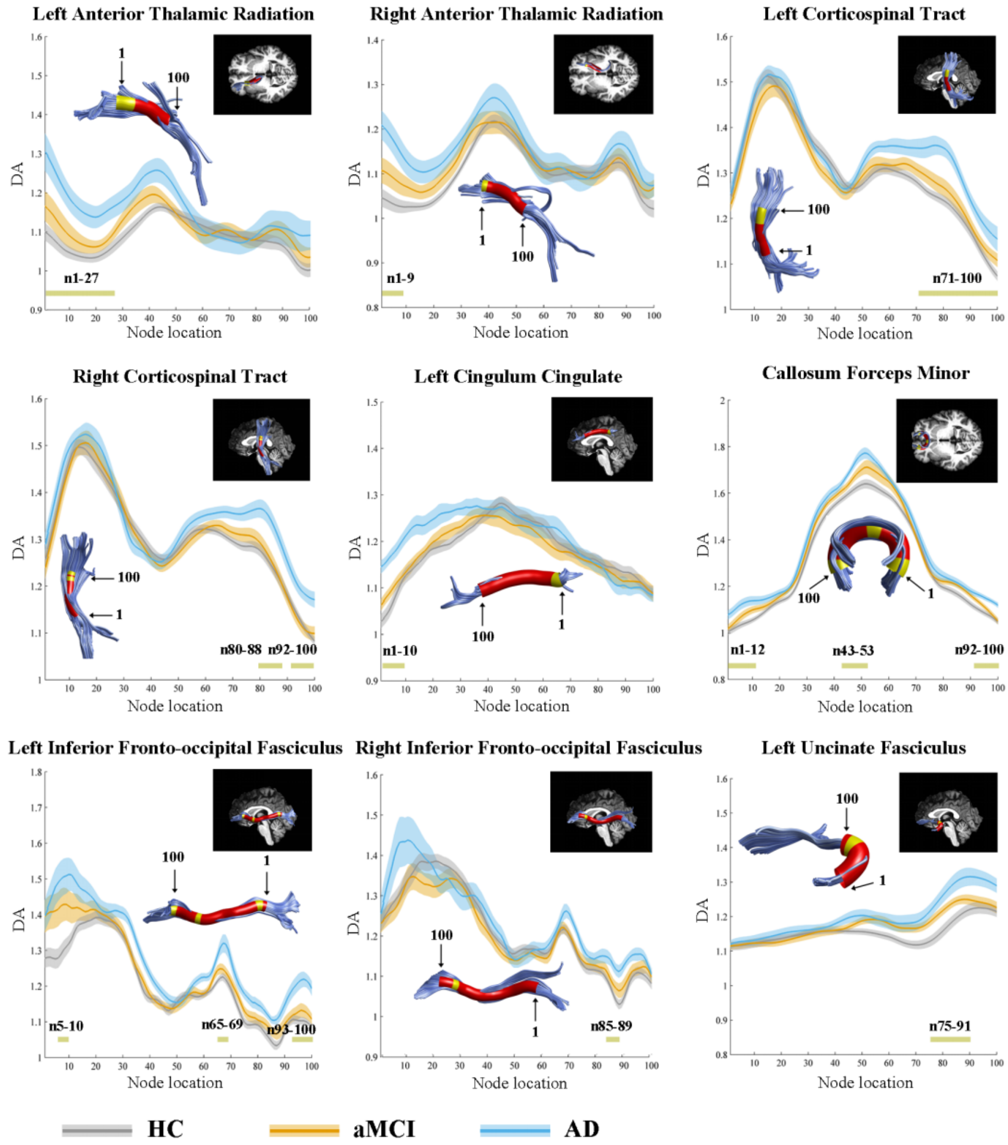

Significantly altered DA values in point-wise of fiber tracts (FDR correction, $p < 0.05$). Yellow color in the white matter tracts represents significantly altered locations. Red color in the white matter tracts represents other locations with no statistical significance. Abbreviation: HC, health control; aMCI, amnestic mild cognitive impairment; AD, Alzheimer's Disease; DA, axial diffusivity.

Supplementary Figure 2

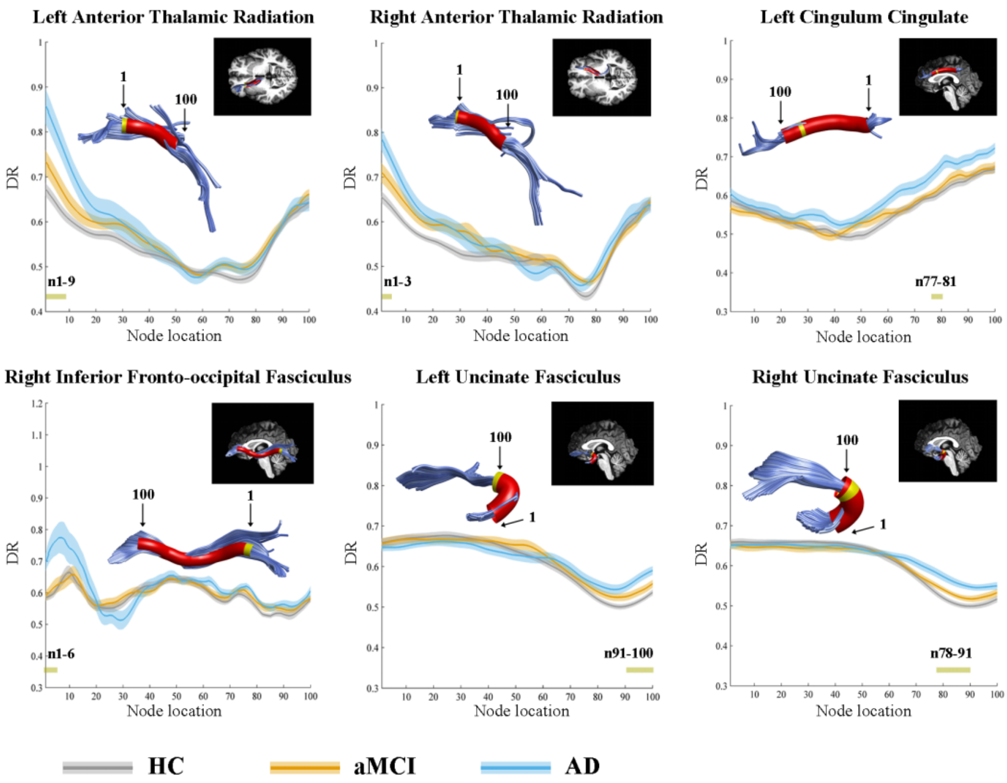

Significantly altered DR values in point-wise of fiber tracts (FDR correction, $p < 0.05$). Yellow color in the white matter tracts represents significantly altered locations. Red color in the white matter tracts represents other locations with no statistical significance. Abbreviation: HC, health control; aMCI, amnestic mild cognitive impairment; AD, Alzheimer's Disease; DR, radial diffusivity.

Supplementary Figure 3

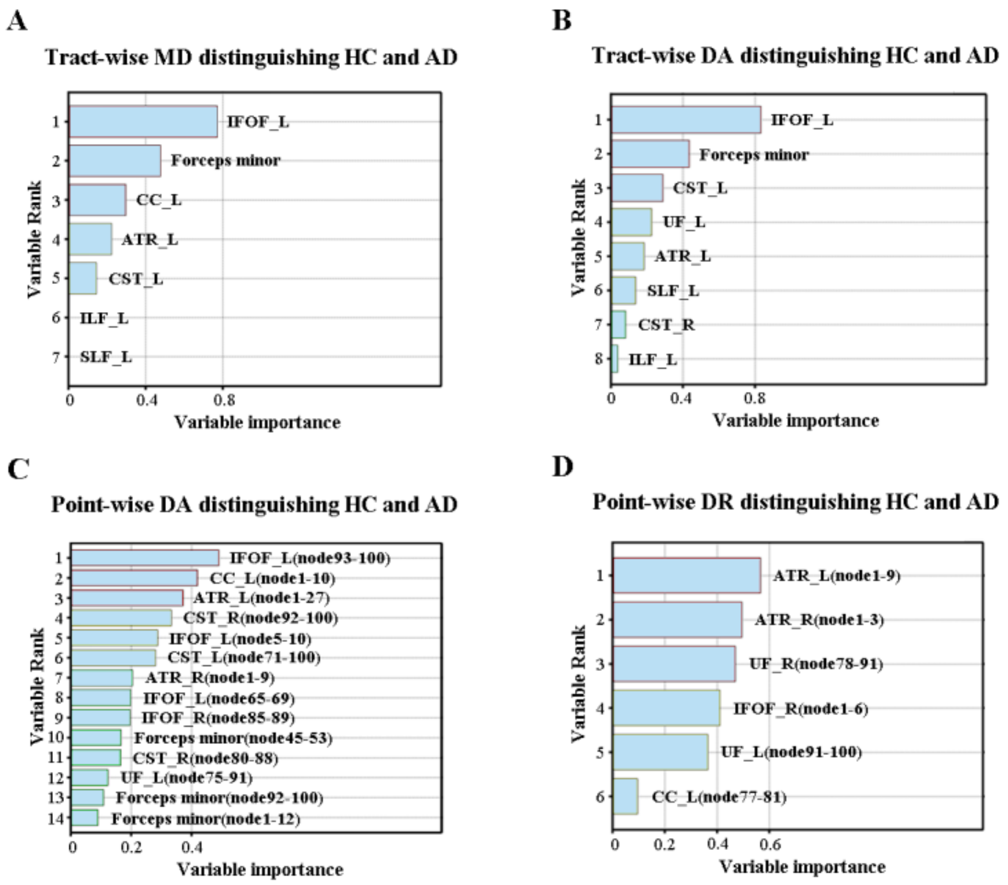

The variable importance of disease diagnosis. (A) The tract-level MD profile distinguished AD from HC with discrimination ability (accuracy = 76.74%; sensitivity = 84.00%; specificity = 66.67%). The left IFOF were the most important tract contributing to AD *VS* HC classification. (B) The tract-level DA measurements distinguished AD from HC with discrimination ability (accuracy = 81.40%; sensitivity = 84.00%; specificity = 77.78%). The left IFOF was the most important tract contributing to AD *VS* HC classification. (C) The point-wise DA measurements distinguished AD from HC with discrimination ability (accuracy = 83.72%; sensitivity = 88.00%; specificity = 83.33%), where the prefrontal component of the left IFOF

was the most important variable. (D) The point-wise DR measurements distinguished AD from HC with discrimination ability (accuracy = 81.40%; sensitivity = 88.00%; specificity = 72.22%). The prefrontal portion of left ATR was the most important variable contributing to AD *VS* HC classification. Abbreviation: HC, health control; aMCI, amnestic mild cognitive impairment; AD, Alzheimer’s Disease; MD, mean diffusivity; DA, axial diffusivity; DR, radial diffusivity; ATR, anterior thalamic radiation; CST, corticospinal tract; CC, cingulum cingulate; IFOF, inferior fronto-occipital fasciculus; ILF, inferior longitudinal fasciculus; SLF, superior longitudinal fasciculus; UF, uncinate fasciculus; R, right; L, left.

Supplementary Table 1

Supplementary Table 1 The identification rate for each of 20 white matter tracts in HC, aMCI and AD

Index	Tract	Total subjects or raw samples (N1)				No. of subjects showing successful tract identification (N2)				Ratio (N2/N1)			
		HC	aMCI	AD	Total	HC	aMCI	AD	Total	HC	aMCI	AD	Total
1	ATR_L	25	26	30	81	25	26	30	81	100.00%	100.00%	100.00%	100.00%
2	ATR_R	25	26	30	81	25	26	30	81	100.00%	100.00%	100.00%	100.00%
3	CST_L	25	26	30	81	25	26	30	81	100.00%	100.00%	100.00%	100.00%
4	CST_R	25	26	30	81	25	26	30	81	100.00%	100.00%	100.00%	100.00%
5	CC_L	25	26	30	81	25	26	30	81	100.00%	100.00%	100.00%	100.00%
6	CC_R	25	26	30	81	25	25	28	78	100.00%	96.15%	93.33%	96.30%
7	CH_L	25	26	30	81	21	13	5	39	84.00%	50.00%	16.67%	48.15%
8	CH_R	25	26	30	81	11	17	18	46	44.00%	65.38%	60.00%	56.79%
9	Forceps major	25	26	30	81	25	26	28	79	100.00%	100.00%	93.33%	97.53%
10	Forceps minor	25	26	30	81	25	26	30	81	100.00%	100.00%	100.00%	100.00%
11	IFOF_L	25	26	30	81	25	26	27	78	100.00%	100.00%	90.00%	96.30%
12	IFOF_R	25	26	30	81	25	25	28	78	100.00%	96.15%	93.33%	96.30%
13	ILF_L	25	26	30	81	25	26	30	81	100.00%	100.00%	100.00%	100.00%
14	ILF_R	25	26	30	81	25	26	28	79	100.00%	100.00%	93.33%	97.53%
15	SLF_L	25	26	30	81	25	26	30	81	100.00%	100.00%	100.00%	100.00%
16	SLF_R	25	26	30	81	25	26	30	81	100.00%	100.00%	100.00%	100.00%
17	UF_L	25	26	30	81	25	26	30	81	100.00%	100.00%	100.00%	100.00%
18	UF_R	25	26	30	81	25	26	30	81	100.00%	100.00%	100.00%	100.00%
19	AF_L	25	26	30	81	23	26	28	77	92.00%	100.00%	93.33%	95.06%
20	AF_R	25	26	30	81	21	26	27	74	84.00%	100.00%	90.00%	91.36%

Abbreviations: HC, health control; aMCI, amnestic mild cognitive impairment; AD, Alzheimer's Disease; ATR, anterior thalamic radiation; CST, corticospinal tract; CC, cingulum cingulate; CH, cingulum hippocampus; IFOF, inferior fronto-occipital fasciculus; ILF, inferior longitudinal fasciculus; SLF, superior longitudinal fasciculus; UF, uncinate fasciculus; AF, arcuate fasciculus; R, right; L, left.

Supplementary Table 2

Supplementary Table 2 Mean FA (× 100) of 16 fiber tracts for HC, aMCI and AD

Index	Tract	Group			*F*	*p*
		HC	aMCI	AD		
1	ATR_L	41.87±2.78	40.98±3.31	41.94±3.77	0.853	0.431
2	ATR_R	43.26±2.55	42.08±3.41	43.12±2.87	0.794	0.457
3	CST_L	60.05±2.80	59.07±2.55	60.06±4.02	0.491	0.614
4	CST_R	60.03±2.69	58.19±2.45	60.09±4.03	1.568	0.217
5	CC_L	45.24±4.14	44.89±3.86	43.95±3.63	0.956	0.390
6	CC_R	40.71±3.92	41.15±4.59	40.90±4.47	0.002	0.998
7	Forceps major	56.54±3.33	55.57±5.74	54.83±4.88	0.575	0.566
8	Forceps minor	49.95±3.28	49.42±3.60	49.29±2.40	0.041	0.960
9	IFOF_L	42.75±2.84	43.08±3.95	43.76±3.80	0.919	0.404
10	IFOF_R	43.95±3.30	43.34±3.39	43.62±4.09	0.071	0.931
11	ILF_L	42.06±3.00	42.68±3.82	41.71±3.25	0.644	0.529
12	ILF_R	39.86±3.54	40.22±3.20	41.74±4.58	1.019	0.367
13	SLF_L	43.29±3.52	43.08±4.93	43.81±3.44	0.088	0.916
14	SLF_R	45.32±3.67	44.27±4.98	45.31±4.54	0.295	0.746
15	UF_L	39.17±2.54	39.65±3.19	40.63±3.97	0.900	0.412
16	UF_R	38.79±2.59	38.91±3.47	37.97±2.24	0.875	0.422

Values are presented as the mean ± standard deviation (SD)

Abbreviation: HC, health control; aMCI, amnestic mild cognitive impairment; AD, Alzheimer's Disease; FA, fractional anisotropy; ATR, anterior thalamic radiation; CST, corticospinal tract; CC, cingulum cingulate; IFOF, inferior fronto-occipital fasciculus; ILF, inferior longitudinal fasciculus; SLF, superior longitudinal fasciculus; UF, uncinate fasciculus; R, right; L, left.

Supplementary Table 3

Supplementary Table 3 Mean DA (× 100) of 16 fiber tracts for HC, aMCI and AD									
**Index**	**Tract**	**Group**			***F***	***p***	***Post hoc analyses***		
		**HC**	**aMCI**	**AD**			**HC *VS* aMCI**	**HC *VS* AD**	**aMCI *VS* AD**
1	ATR_L	108.54±4.38	110.88±7.59	115.36±8.97	5.478	0.006*	0.761	0.003*	0.008*
2	ATR_R	110.26±4.74	112.09±7.31	115.12±8.72	2.710	0.075	--	--	--
3	CST_L	130.72±4.06	130.76±5.77	134.80±4.29	5.663	0.006*	0.740	0.007*	0.003*
4	CST_R	130.87±4.02	131.02±5.22	134.53±3.86	4.278	0.018*	0.656	0.022*	0.008*
5	CC_L	118.09±4.42	117.88±6.53	120.54±4.95	1.364	0.263	--	--	--
6	CC_R	110.55±3.71	112.30±8.18	112.83±6.49	0.584	0.561	--	--	--
7	Forceps major	156.91±7.55	159.24±10.52	158.87±8.82	0.208	0.813	--	--	--
8	Forceps minor	130.47±5.59	133.75±5.87	137.13±5.52	7.196	0.002*	0.139	<0.001*	0.022*
9	IFOF_L	120.67±3.68	123.46±6.87	128.21±4.34	11.096	<0.001*	0.035*	<0.001*	0.010*
10	IFOF_R	121.42±3.56	120.51±5.87	124.13±6.54	2.553	0.086	--	--	--
11	ILF_L	121.68±4.92	122.20±6.93	125.82±6.67	4.019	0.023*	0.631	0.009*	0.030*
12	ILF_R	115.56±4.99	116.42±7.50	117.23±6.28	0.593	0.556	--	--	--
13	SLF_L	108.85±5.00	109.15±5.41	113.48±6.30	4.292	0.018*	0.872	0.010*	0.015*
14	SLF_R	111.99±4.01	111.43±7.85	115.46±6.49	1.833	0.169	--	--	--
15	UF_L	115.36±3.34	117.37±3.53	119.92±5.74	6.780	0.002*	0.074	<0.001*	0.056
16	UF_R	114.37±4.20	114.20±4.28	114.67±5.27	0.007	0.993	--	--	--

Values are presented as the mean ± standard deviation (SD)

* indicates a statistical difference between groups, p < 0.05

Abbreviation: HC, health control; aMCI, amnestic mild cognitive impairment; AD, Alzheimer's Disease; DA, axial diffusivity; ATR, anterior thalamic radiation; CST, corticospinal tract; CC, cingulum cingulate; IFOF, inferior fronto-occipital fasciculus; ILF, inferior longitudinal fasciculus; SLF, superior longitudinal fasciculus; UF, uncinate fasciculus; R, right; L, left.

Supplementary Table 4

Supplementary Table 4 Mean DR (× 100) of 16 fiber tracts for HC, aMCI and AD									
**Index**	**Tract**	**Group**			***F***	***p***	***Post hoc analyses***		
		**HC**	**aMCI**	**AD**			**HC *VS* aMCI**	**HC *VS* AD**	**aMCI *VS* AD**
1	ATR_L	54.65±2.61	56.82±4.15	58.40±6.46	2.656	0.078	--	--	--
2	ATR_R	53.69±2.63	56.05±4.61	56.59±5.35	1.160	0.320	--	--	--
3	CST_L	44.31±2.86	45.56±2.30	45.56±4.00	0.490	0.615	--	--	--
4	CST_R	44.44±2.65	46.50±2.25	45.58±4.14	1.001	0.373	--	--	--
5	CC_L	56.09±3.79	56.63±4.19	59.80±4.12	6.357	0.003*	0.703	0.002*	0.005*
6	CC_R	57.26±3.80	57.87±3.14	58.62±4.54	0.845	0.435	--	--	--
7	Forceps major	57.46±7.01	59.91±10.52	60.47±9.32	0.477	0.623	--	--	--
8	Forceps minor	54.01±4.19	56.08±5.40	57.58±4.02	2.521	0.089	--	--	--
9	IFOF_L	60.05±3.12	61.26±5.70	62.85±5.10	1.804	0.173	--	--	--
10	IFOF_R	58.97±3.56	59.62±4.73	61.49±6.72	1.996	0.145	--	--	--
11	ILF_L	61.14±2.58	60.84±5.45	63.67±4.78	3.366	0.041*	0.682	0.042*	0.017*
12	ILF_R	60.96±3.75	61.12±3.67	59.49±5.42	0.470	0.628	--	--	--
13	SLF_L	55.14±2.79	55.45±3.45	55.90±3.40	0.692	0.505	--	--	--
14	SLF_R	54.48±3.31	55.12±3.70	55.38±4.03	0.344	0.710	--	--	--
15	UF_L	61.10±2.95	61.92±3.82	61.75±3.99	0.349	0.707	--	--	--
16	UF_R	60.67±2.27	60.66±3.63	61.91±3.27	1.047	0.357	--	--	--

Values are presented as the mean ± standard deviation (SD)

* indicates a statistical difference between groups, p < 0.05

Abbreviation: HC, health control; aMCI, amnestic mild cognitive impairment; AD, Alzheimer's Disease; DR, radial diffusivity; ATR, anterior thalamic radiation; CST, corticospinal tract; CC, cingulum cingulate; IFOF, inferior fronto-occipital fasciculus; ILF, inferior longitudinal fasciculus; SLF, superior longitudinal fasciculus; UF, uncinate fasciculus; R, right; L, left.
